# The effect of pre-diagnostic vitamin D supplementation on cancer survival in women: a cohort study within the UK Clinical Practice Research Datalink

**DOI:** 10.1186/s12885-015-1684-0

**Published:** 2015-10-12

**Authors:** Mona Jeffreys, Maria Theresa Redaniel, Richard M. Martin

**Affiliations:** 1 School of Social and Community Medicine, University of Bristol, Canynge Hall, 39 Whatley Road, Bristol, BS8 2PS UK; 2 NIHR CLAHRC West, 9th Floor, Whitefriars, Lewins Mead, Bristol, BS1 2NT UK; 3 Medical Research Council/University of Bristol Integrated Epidemiology Unit, University of Bristol, Bristol, UK; 4 University of Bristol/University Hospitals Bristol NHS Foundation Trust National Institute for Health Research Bristol Nutrition Biomedical Research Unit, University of Bristol, Bristol, UK

**Keywords:** Vitamin D, Cancer, Cancer survival, Confounding by indication

## Abstract

**Background:**

There remains uncertainty in whether vitamin D status affects cancer survival. We investigated whether vitamin D (± calcium) supplementation affects cancer survival in women.

**Methods:**

Participants were women aged ≥55 years identified from the UK Clinical Practice Research Datalink (CPRD) with a first diagnosis of breast, colorectal, lung, ovarian or uterine cancer between 2002 and 2009, and at least 5 years of CPRD data prior to diagnosis. Cox proportional hazards were used to estimate hazard ratios (HR) and 95 % confidence intervals (CI) of the relationship between pre-diagnostic vitamin D supplementation and all-cause mortality. To avoid confounding by indication, the primary analysis compared women with 3+ to 1–2 (but no more) vitamin D prescriptions. Models were adjusted for pre-diagnostic body mass index, smoking, alcohol and deprivation. A sensitivity analysis excluded supplements prescribed in the year prior to diagnosis.

**Results:**

Exposure to 3 or more versus 1 to 2 prescriptions of vitamin D was not associated with survival from any of the cancers studied. Any vitamin D prescription, compared to never having been prescribed one, was associated with a better survival from breast cancer (HR 0.78, 95 % CI 0.70 to 0.88). The sensitivity analysis suggested a possible detrimental effect of vitamin D supplementation on lung cancer outcomes (HR for 3 versus 1 or 2 prescriptions 1.22 (95 % CI 0.94 to 1.57); HR for any versus no prescriptions 1.09 (0.98 to 1.22)).

**Conclusions:**

We found no evidence that vitamin D supplementation is associated with survival among women with cancer. Previous observational findings of beneficial effects of vitamin D supplementation on cancer survival may be confounded.

## Background

The benefits of vitamin D have received much attention, deriving primarily from observational data, which suggest that low vitamin D status is associated with higher mortality [1, 2]. Key to understanding this association is to determine whether low vitamin D levels cause premature death, or whether the vitamin D levels are a consequence of poor health. If vitamin D is simply a marker of health status, supplementation is unlikely to have a direct benefit on mortality. If the association is causal, then vitamin D supplementation is likely to be of some benefit in reducing mortality. An individual patient data meta-analysis of randomised controlled trials (RCTs) found lower all-cause mortality in patients receiving vitamin D and calcium compared to placebo [3], and a meta-analysis of three studies also demonstrated this in relation to cancer mortality in patients with cancer [4].

Observational evidence relating vitamin D levels to cancer survival is strongest for colorectal cancer, in which ecological and individual level data consistently show better survival in people with higher vitamin D levels [5]. However, a review concluded that there is no strong nor consistent evidence that vitamin D reduces the risk of ovarian cancer mortality [6].

There is very limited evidence on this topic from RCTs. Follow-up of the Women’s Health Initiative trial found a suggestion of a beneficial effect of vitamin D supplementation on cancer mortality (hazard ratio (HR) 0.89, 95 % confidence interval (CI): 0.77 to 1.03) [7]. Follow-up of participants in the RECORD trial found no effect of vitamin D supplementation on cancer mortality in an intention to treat analysis, and a possible beneficial effect in an analysis adjusted for compliance [8]. When the two trials were pooled, there was a suggestion of a beneficial effect of vitamin D on colorectal cancer mortality (risk ratio 0.78; 95 % CI: 0.52 to 1.17) [5].

Vitamin D and calcium supplements are routinely given to older women to prevent osteoporotic fractures. Current vitamin D supplementation guidelines recommend daily supplements containing 10 mcg amongst people aged 65 years or over, or who are not exposed to much sun (for example, those who cover up their skin for cultural reasons, who are housebound on confined indoors for long periods, or those who have darker skin) [9]. Approximately 5 % of women over the age of 60 in the UK have received at least one year’s worth of supplements [10]. Whether these supplements affect survival following a cancer diagnosis, remains unclear.

In the absence of randomised evidence, alternatives are required to address issues of causality. Observational studies of vitamin D supplementation are prone to confounding by indication, whereby an apparent association between vitamin D and an outcome is due to characteristics of those prescribed vitamin D (including the indication for prescription), not vitamin D itself [11]. The association of vitamin D and survival may be confounded if women given a prescription might be manifesting symptoms that are indicative of cancer and are predictive of prognosis or survival, but have been mistaken for (e.g. bone pain) or cause (e.g. anorexia affecting nutrient intake or frailty impeding ability to go outdoors) vitamin D deficiency. The presence of osteoporosis, which is related to low estrogen levels, may also influence vitamin D supplement use and breast cancer prognosis. The association may also be confounded if manifesting symptoms cause discontinuation of vitamin D supplementation. To address this, we conducted an analysis with an *a priori* comparison of women who discontinue compared to those who continue with prescribed vitamin D supplements. We have previously reported no strong link between continuing vs. discontinuation vitamin D supplementation and the risk of breast, colorectal, lung, ovarian or uterine cancer among women with cancer in the UK Clinical Practice Research Datalink (CPRD, formerly the General Practice Research Database (GPRD)) [12]. Here we report on the effect of prediagnostic prescribed vitamin D supplements on all cause mortality in a cohort of women with cancer.

## Methods

We conducted an analysis of cancer survival within the CPRD, a database of anonymised, longitudinal medical records of patients registered with contributing primary care practices across the UK (CPRD, personal communication). As of September 2014, the CPRD database covers approximately 8.8 % of the UK population from 684 GP practices (CPRD, personal communication). There are research standard quality data for 13.58 M patients in CPRD, of which 5.69 M are active (still alive and registered with the GP practice). Data is said to be of research standard quality if the record satisfies pre-specified minimum data quality criteria that include thresholds for practice death recording and missing data [13, 14]. Access to CPRD data was granted by the CPRD-Independent Scientific Advisory Committee (CPRD-ISAC), an advisory body established to provide advice on request to access data provided by the CPRD [15]. Use of anonymised CPRD data is approved by the Trent Multi-Centre Research Ethics Committee (05/MRE/04/87).

Participants were women aged 55 years or over at the time of a first diagnosis of breast, colorectal, lung, ovarian or uterine cancer between 2002 and 2011, representing post-menopausal women. This analysis was limited to women as the focus of the grant application was common cancers in women. Codes used to identify participants were listed by the authors, and supplemented by those suggested by CPRD staff. These are available on request from the authors. Further inclusion criteria were: the practice having at least 5 years’ worth of research standard quality data prior to the date of cancer diagnosis. Follow-up extended from the date of cancer diagnosis to the earliest of: death, leaving the practice, or the final date of data collection, defined on a practice level. Information on the cause of death was not available in our dataset and we only present survival from all causes.

A total of 21,932 women were diagnosed with one of the five cancers of interest during the study period. Two women who were recorded as dying, one and 3 months respectively, prior to their cancer diagnosis were excluded. A further 365 women who died on their date of diagnosis were also excluded, leaving 21,565 women for analysis (11,112 women with breast cancer; 4122 with colorectal cancer; 3352 with lung cancer and 2979 with gynaecological cancer).

Women were classified as either having received none, 1–2 (reference) or 3 or more prescriptions for vitamin D ± calcium (BNF Chapters 9.6.4 and 9.5.1.1) in the 5 years prior to cancer diagnosis. Associations of vitamin D supplementation with survival from each cancer were determined using Cox proportional hazards models. Robust standard errors were used to account for clustering at a practice level. Adherence to the proportional hazards assumption was tested graphically and empirically, using Schoenfeld residuals. Basic models were adjusted for the following covariates: age (as a continuous variable, and in six 5-year age bands, from 55 to 59 to the upper age band being 80 years and over), period of diagnosis (calendar years 2002–2003, 2004–2005, 2006–2007, 2008–2009, 2010–2011). Multivariable models also included smoking (never, current and ex), alcohol consumption (any vs none/ex status), body mass index (underweight: <18.5, normal: 18.5–24.9, overweight: 25–29.9 and obese: ≥30 kg/m^2^) and deprivation, measured using the Index of Multiple Deprivation (IMD) score. The IMD is a small area level measure of socio-economic status (based on patients’ area of residence at the time of diagnosis), which is computed from a number of social and economic indicators (housing, employment, income, access to services, education and skills, crime, living environment) [16]. Approximately half the CPRD practices consented to their patients’ addresses being linked to an IMD score. Study-specific quintiles of this score were used in the analysis. Missing data for all potential confounders were retained in the analysis, coded to a separate category. There were no consistent prescribing patterns by season in the years included in the study period (data not shown) and this was not adjusted for in the analysis.

To assess the effect of altered prescribing of vitamin D supplements around the time of a cancer diagnosis, a sensitivity analysis was undertaken, excluding women who received their first prescription of vitamin D (± calcium) within a year prior to diagnosis.

We have also conducted a sensitivity analysis only including women aged 60 and over. This strategy was used to exploit the free prescription coverage for these women, who may be less likely to be consuming vitamin D from over the counter sources. In this age older group, therefore, misclassification of vitamin D supplementation may be less likely to distort the results. We found no difference between this analysis and our results including the entire sample (data not shown).

## Results

Of the 21,932 women included in the analysis, 18,998 (88 %) did not have any vitamin D prescription during the period from their GP practice becoming up to research standard and the date of their cancer diagnosis. Nine percent (*n* = 1906) had three or more prescriptions and 3 % (*n* = 661) had one or two prescriptions only. The median duration of intake was 56 days (interquartile range, IQR, 30–100) for patients with 1–2 prescriptions and 504 days (IQR 240–1050) for patients with 3 or more prescriptions. There was a strong relationship between period of diagnosis and vitamin D prescription, with the proportion of women having had three or more prescriptions rising from 4 % in 2002–03 to 13 % in 2010–11.

Table 1 shows the baseline characteristics of the study cohort. We identified 11,112 breast, 4122 colorectal, 3352 lung and 2979 gynaecological (ovarian and uterine) cancer cases. The vast majority (97 %) of those taking supplements were prescribed vitamin D in combination with calcium rather than alone. The median length of follow-up was 30.4 months (inter quartile range 1 to 115 months). During this follow-up time, there were 7736 deaths (2103 in women with breast cancer; 1726 in women with colorectal cancer; 2756 in women with lung cancer and 1151 in women with a gynaecological cancer).

**Table 1:** Characteristics of women with specific cancers, identified through the General Practice Research Datalink (2002–2011)

	Breast		Colorectal		Lung		Gynaecological^a^	
	*N* = 11,112		*N* = 4122		*N* = 3352		*N* = 2979	
	n	%	n	%	n	%	n	%
Supplementation								
None	9952	89.6	3566	86.5	2793	83.3	2687	90.2
1–2 prescriptions	318	2.9	130	3.2	141	4.2	72	2.4
3+ prescriptions	842	7.6	426	10.3	418	12.5	220	7.4
Age group								
<60	2019	18.2	324	7.9	249	7.4	484	16.3
60–64	2213	19.9	441	10.7	438	13.1	586	19.7
65–69	1888	17.0	564	13.7	516	15.4	557	18.7
70–74	1427	12.8	648	15.7	628	18.7	514	17.3
75–79	1339	12.1	761	18.5	651	19.4	360	12.1
80 and above	2226	20.0	1384	33.6	870	26.0	478	16.1
Period of diagnosis								
2002–03	1809	16.3	587	14.2	453	13.5	438	14.7
2004–05	2582	23.2	920	22.3	736	22.0	670	22.5
2006–07	3182	28.6	1175	28.5	981	29.3	841	28.2
2008–09	3289	29.6	1308	31.7	1067	31.8	951	31.9
2010–11	250	2.3	132	3.2	115	3.4	79	2.7
Vital status at follow-up								
Alive	9009	81.1	2396	58.1	596	17.8	1828	61.4
Dead	2103	18.9	1726	41.9	2756	82.2	1151	38.6
Smoking								
Never	6793	61.1	2539	61.6	523	15.6	1936	65.0
Current	1426	12.8	444	10.8	1399	41.7	308	10.3
Ex	2559	23.0	1020	24.8	1383	41.3	654	22.0
Missing	334	3.0	119	2.9	47	1.4	81	2.7
Alcohol								
None/ex	2569	23.1	1081	26.2	984	29.4	716	24.0
Any	7383	66.4	2533	61.5	1980	59.1	1930	64.8
Missing	1160	10.4	508	12.3	388	11.6	333	11.2
BMI^b^								
Underweight	155	1.4	118	2.9	221	6.6	41	1.4
Normal	3576	32.2	1451	35.2	1314	39.2	788	26.5
Overweight	3498	31.5	1227	29.8	883	26.3	877	29.4
Obese	2656	23.9	804	19.5	539	16.1	951	31.9
Missing	1227	11.0	522	12.7	395	11.8	322	10.8
Deprivation level^c^								
Quintile 1 (deprived)	913	8.2	391	9.5	508	15.2	270	9.1
Quintile 2	1064	9.6	411	10.0	362	10.8	268	9.0
Quintile 3	1152	10.4	383	9.3	255	7.6	305	10.2
Quintile 4	1181	10.6	403	9.8	204	6.1	319	10.7
Quintile 5 (affluent)	1217	11.0	369	9.0	204	6.1	313	10.5
Missing	5585	50.3	2165	52.5	1819	54.3	1504	50.5

^a^Gynaecological cancers included 1372 women with ovarian cancer, 1599 women with uterine cancer and 8 women with other unspecificed gynaecological cancers

^b^Underweight: <18.5 kg/m^2^; normal weight: 18.5 to <25 kg/m^2^; overweight 25 to <30 kg/m^2^; obese > =30 kg/m^2^

^c^Study-specific quintiles of the Index of Multiple Deprivation (IMD) score, based on patient’s address

Exposure to three or more prescriptions of vitamin D was not associated with survival from any of the cancers that we studied, compared with 1–2 prescriptions (Table 2). This effect remained the same after adjustment for BMI, smoking status, alcohol drinking and level of deprivation. For breast, colorectal and gynaecological cancers, having been prescribed a vitamin D supplement was associated with lower mortality than not having been prescribed a supplement, although this only reached conventional levels of statistical significance in women with breast cancer.

**Table 2:** The association of vitamin D and calcium supplementation with survival from selected cancers in women

	Deaths	Cases	Person-	Basic model		Adjusted model	
			Years	HR	95 % CI	HR	95 % CI
Breast cancer							
3+ prescriptions	228	842	2408.5	1.01	0.79 to 1.29	1.02	0.79 to 1.32
1–2 prescriptions	86	318	974.9	1		1	
Any	314	1160	3383.3	0.80	0.71 to 0.90	0.78	0.70 to 0.88
None	1789	9952	38168.7	1		1	
Colorectal cancer							
3+ prescriptions	191	426	929.7	0.82	0.61 to 1.10	0.81	0.59 to 1.11
1–2 prescriptions	61	130	282.1	1		1	
Any	252	556	1203.8	0.91	0.79 to 1.04	0.90	0.78 to 1.04
None	1474	3566	10090.6	1		1	
Lung cancer							
3+ prescriptions	323	418	384.0	0.91	0.73 to 1.12	0.86	0.70 to 1.07
1–2 prescriptions	120	141	134.1	1		1	
Any	443	559	518.1	1.05	0.96 to 1.16	1.06	0.96 to 1.17
None	2313	2793	2954.1	1		1	
Gynaecologic cancer							
3+ prescriptions	98	220	538.1	0.74	0.49 to 1.10	0.84	0.59 to 1.30
1–2 prescriptions	36	72	152.7	1		1	
Any	134	292	690.9	0.87	0.72 to 1.05	0.89	0.73 to 1.07
None	1017	2687	8033.1	1		1	

The basic model is adjusted for age and period; the adjusted model is further adjusted for smoking, alcohol, BMI and area-level deprivation

In the sensitivity analysis (Table 3), excluding all supplements prescribed in the 1 year prior to diagnosis did not materially alter the interpretation of the results, although there was a suggestion in these analyses that vitamin D supplementation may be associated with a higher risk of mortality in women with lung cancer.

**Table 3:** The association of vitamin D and calcium supplementation with survival from selected cancers in women: sensitivity analysis

	Deaths	Cases	Person-	Basic model		Adjusted model	
			Years	HR	95 % CI	HR	95 % CI
Breast cancer							
3+ prescriptions	176	670	1890.8	1.05	0.79 to 1.40	1.08	0.81 to 1.44
1–2 prescriptions	60	223	685.3	1		1	
Any	236	893	2576.0	0.81	0.71 to 0.92	0.80	0.70 to 0.91
None	1867	10,219	38976.0	1		1	
Colorectal cancer							
3+ prescriptions	148	332	708.2	1.08	0.76 to 1.54	1.06	0.74 to 1.52
1–2 prescriptions	45	111	247.8	1		1	
Any	193	443	956.0	0.96	0.82 to 1.12	0.95	0.82 to 1.11
None	1533	3679	10338.3	1		1	
Lung cancer							
3+ prescriptions	251	324	283.8	1.22	0.95 to 1.56	1.22	0.94 to 1.57
1–2 prescriptions	87	109	119.3	1		1	
Any	338	433	403.0	1.09	0.98 to 1.21	1.09	0.98 to 1.22
None	2418	2919	3069.1	1		1	
Gynaecologic cancer							
3+ prescriptions	78	163	372.6	1.27	0.76 to 2.12	1.24	0.71 to 2.18
1–2 prescriptions	21	61	145.6	1		1	
Any	99	224	518.1	0.94	0.75 to 1.17	0.95	0.76 to 1.19
None	1052	2755	8205.8	1		1	

This sensitivity analysis excludes all supplements prescribed in the year prior to cancer diagnosis

The basic model is adjusted for age and period; the adjusted model is further adjusted for smoking, alcohol, BMI and area-level deprivation

## Discussion

This study, designed to address the issue confounding by indication [11] and reverse causality, found that pre-diagnostic vitamin D supplementation has little effect on survival in women with one of four major cancers. Furthermore, our results highlight the need for caution in interpreting observational data of vitamin D supplementation and cancer survival, given the marked protective effect on mortality seen in women with breast cancer who are prescribed supplements, compared with those never prescribed supplements.

A high validity of using cancer diagnoses as recorded in the GPRD has previously been reported [17]. Even if some cases of cancers were omitted erroneously from the dataset, it is unlikely that this would introduce any selection bias into the study, since the association between vitamin D supplementation and mortality is unlikely to differ between those included and those excluded. In the validation study, the median time between diagnosis in GPRD and in the cancer registry data was 11 days [17], suggesting that our sensitivity analysis of excluding a full year prior to the date of diagnosis would be sufficiently sensitive.

A systematic review of the validity of reporting in GPRD found just one validation study, on sudden death, which was well reported [18]. Potential under-ascertainment of outcome may have diluted our effect, but is unlikely to have been an important source of bias in this study.

We were limited by our measure of supplementation; in particular, we had no information on vitamin D bought over the counter. Some women who had not received any vitamin D prescription may have bought vitamin D. However, it is unlikely that women who had received a prescription would instead buy vitamin D, since prescriptions for women are free after age 60 years. Therefore, this should have had minimal impact on our *a priori* results. In CPRD, dosage is reported, but instructions for use are not complete, precluding a calculation of average daily dose, or equivalent. We have previously reported a moderate degree of correlation between number of prescriptions and duration of intake (r^2^ = 0.66, *p* < 0.01) [12]. Any exposure misclassification is likely to be non-differential, and therefore have diluted our results towards the null effect, rather than to have caused measurement bias.

We also do not have any information on adherence to vitamin D supplementation in the UK. Previous studies have shown that among elderly female hip fracture patients, compliance to recommended supplements was low (28.9 %), but that it can be increased through written recommendations in the hospital discharge letter [19]. In the UK, current guidelines include recommendations to improve vitamin D access for women over the age of 65, including free prescriptions to women over 60. These guidelines may keep non-adherece to a lower level.

We were unable to adjust for key clinical determinants of survival, such as stage of disease. There is some evidence that low levels of vitamin D may be associated with faster progression of cancer [20]. For example, in the Health, Eating, Activity, and Lifestyle study, stage of disease predicted vitamin D levels, independent of other potential confounders [21].

Other studies have demonstrated that vitamin D levels may be related to adverse prognostic indicators, such as tumour size (but not grade) [22] and hormone receptor profiles with poorer prognosis (but not tumour size or invasiveness) [23]. Although we could not test this, it seems plausible that the lack of adjustment of key prognostic markers will not have affected our inferences of an effect of vitamin D on survival to a strong degree. Given that our exposure and reference groups differed by discontinuation rather than by initiation of supplementation, it seems unlikely that the two groups would differ by key determinants of survival. Indeed, this has been empirically shown in a study of ovarian cancer [24].

It is worth noting that most studies looking at cancer progression used blood levels of vitamin D as its measure, and might not be directly comparable to our study using vitamin D prescriptions. Vitamin D supplementation might not correlate with serum levels of vitamin D, since sun exposure and intake of vitamin D food sources affects vitamin D serum levels. Nevertheless, vitamin D from sun exposure is limited in the UK, as much of the country is situated above the latitude that permits optimal vitamin D synthesis, particularly during fall and winter. The elderly, such as the women in our study population, also spend relatively large amounts of time indoors, have reduced dermal capacity to synthesize vitamin D and were more likely to use sun protection when outdoors.

More comparable clinical trials have shown inconsistent results and it remains unclear whether the post-diagnostic supplementation of patients with cancer can improve survival. Three trials (summarised in [25]) of vitamin D supplementation in men with prostate cancer provided conflicting results; after the promsing ASCENT trial, the ASCENT-II trial was stopped early, due to a higher rate of death in the supplemented group. Ongoing trials are evaluating the role that vitamin D may play on survival in patients with metastatic breast cancer, chronic lymphoid leukaemia and melanoma [26]. Moreover, further research is required for other outcomes. For example, initial results show a possible role vitamin D supplementation may play in reducing aromatase inhibitor-induced joint symptoms [27] and loss of bone density [20] in women with breast cancer.

## Conclusion

In conclusion, in this population-based study in women in the UK, our results do not support any association between longer compared to short vitamin D supplementation and beneficial survival from breast, colorectal, lung, ovarian or uterine cancers. Women who had been prescribed a vitamin D supplement exhibited better survival. We suggest that previous observational data may have been subject to confounding by indication.
